# Tailoring Oncofertility to Breast Cancer Subtype: A Systematic Review of Fertility Preservation Strategies in Premenopausal Women

**DOI:** 10.3390/cancers18121896

**Published:** 2026-06-10

**Authors:** Maryam Garba Oloriegbe, Olena Bolgova, Rasha Alissa, Aliaa Abdelmeguid, Hamida Garba Oloriegbe, Umaiza Rehan, Volodymyr Mavrych

**Affiliations:** College of Medicine, Alfaisal University, Riyadh 11533, Saudi Arabia

**Keywords:** fertility preservation, breast cancer subtypes, controlled ovarian stimulation, GnRH agonists, oncofertility

## Abstract

Introduction: Breast cancer is the most common malignancy in women of reproductive age, and treatment advances have heightened the importance of fertility preservation (FP) for young patients. Despite heterogeneity across subtypes—hormone receptor-positive (HR+), HER2-positive, triple-negative (TNBC), and BRCA1/2-associated—existing guidelines lack subtype-specific FP guidance. Methods: This systematic review compared FP strategies across subtypes, identified subtype-specific challenges, and proposed pathways toward precision oncofertility care. PubMed, Scopus, and Web of Science were searched following PRISMA guidelines for English-language studies from 2004 to 2024. Results: After screening 1837 records, 19 studies met eligibility criteria (2 RCTs, 17 cohort studies); 11 of 17 non-randomized studies were at low overall risk of bias, and 6 at moderate risk due to confounding. Discussion: COS using letrozole- or tamoxifen-modified protocols was feasible, yielding 8–14 mature oocytes per cycle with reduced estradiol exposure suitable for HR+ disease. Evidence was strongest for HR+ patients; TNBC and HER2+ data were more limited, with some studies noting reduced ovarian reserve. GnRH agonists during chemotherapy reduced ovarian failure rates and improved post-treatment recovery, most consistently in hormone receptor-negative disease. BRCA1/2 carriers showed broadly comparable FP outcomes to non-carriers, though BRCA1-positive patients had modestly reduced oocyte yields in some studies with inconsistent results. Conclusions: Among studies with medium-term follow-up (3–5.5 years), no significant increase in recurrence or mortality attributable to FP was identified; long-term data beyond 5 years remain sparse. Substantial heterogeneity precluded meta-analysis; all synthesis is narrative. Standardized outco reporting and larger prospective subtype-stratified studies are required to establish precision oncofertility recommendations.

## Simple Summary

Breast cancer is the most common cancer in women worldwide, and its diagnosis in young women of reproductive age is increasingly frequent. Modern treatments have dramatically improved survival, but many of these therapies damage the ovaries and threaten future fertility, which can be profoundly distressing for young survivors. Fortunately, established strategies exist to preserve fertility before treatment begins, primarily through freezing eggs or embryos after a short course of hormonal stimulation. This review analyzed 19 studies examining fertility preservation outcomes in young women with breast cancer. The evidence shows that fertility preservation is feasible and effective, with most women responding well to ovarian stimulation. Modified protocols using letrozole appear safe in hormone-sensitive disease. Genetic mutations such as BRCA1/2 may reduce egg yield but do not preclude preservation. Based on currently available evidence (predominantly retrospective observational data with mostly short- to medium-term follow-up), fertility preservation does not appear to worsen cancer outcomes, though definitive long-term reassurance requires prospective studies with extended follow-up. Decisions should be individualized through early specialist counseling.

## 1. Introduction

Breast cancer is the most prevalent malignancy among women worldwide. Annually, around 2.3 million new cases occur, with considerable regional and socioeconomic variation in incidence, mortality, and survival rates [1,2]. The incidence rates among individuals of reproductive age are steadily rising, exacerbating challenges with fertility preservation and long-term quality of life [3]. Multimodal therapeutic advances, including neoadjuvant chemotherapy, targeted therapies, CDK4/6 inhibitors, immunotherapy, and precision oncology, have improved 5-year survival rates to over 90%, markedly increasing the number of young survivors [4,5]. However, these therapies (particularly gonadotoxic chemotherapy with alkylating agents and extended endocrine therapy) threaten ovarian reserve and future fertility, making preservation strategies an urgent clinical imperative [6,7]. For reproductive-age survivors, fertility preservation has become crucial for maintaining quality of life, addressing profound psychosocial distress caused by treatment-induced infertility and premature ovarian insufficiency [6].

To mitigate the gonadotoxic effects of breast cancer therapies, established fertility preservation strategies include embryo and oocyte cryopreservation, which typically require 10–14 days of controlled ovarian stimulation (COS) with gonadotropins, often combined with aromatase inhibitors like letrozole to minimize estrogen exposure in hormone receptor-positive disease [8]. Other options include ovarian tissue cryopreservation, a surgical technique that enables immediate treatment initiation without a delay for stimulation. Investigational and adjunctive approaches, such as in vitro maturation of oocytes, ovarian transposition, and modified stimulation protocols, can expand options for patients requiring urgent oncologic treatment or those with contraindications to standard methods [9,10]. The choice of fertility preservation should be personalized, primarily considering the patient’s age, urgency, the planned treatment’s gonadotoxicity, the nature of the disease, and the time available before therapy starts [8]. Both ASCO and ESMO guidelines recommend urgent, multidisciplinary referral to a fertility specialist and individualized counseling before gonadotoxic therapy begins [11,12]. However, it is important to note the distinction between gamete/embryo cryopreservation, which directly preserves reproductive material, and GnRH agonist use, which protects ovarian function but does not constitute an equivalent FP method; evidence supporting the latter, particularly for live birth outcomes, remains limited.

Breast cancer subtypes differ substantially in biology, prognosis, and treatment. Hormone receptor–positive (ER+/PR+) tumors are typically luminal, often indolent but prone to late recurrences, and managed with long-course endocrine therapy (5–10 years) and CDK4/6 inhibitors in selected high-risk cases [13]. HER2-positive disease is more aggressive but now highly treatable with chemotherapy combined with HER2-targeted therapy, most commonly trastuzumab with or without pertuzumab and standard anthracycline/taxane-based backbones [13]. Triple-negative breast cancer (TNBC) often presents with high-grade, basal-like biology and an early peak in relapse risk, treated with anthracycline–taxane regimens, frequently incorporating platinum and immunotherapy in the neo/adjuvant setting [13,14]. Germline BRCA-associated breast cancer carries a higher lifetime risk and a younger age of onset; systemic therapy commonly includes platinum agents and PARP inhibitors [14,15]. These differences necessitate tailored FP strategies: for ER+/PR+ disease, tamoxifen/letrozole-based COS protocols minimize estrogen exposure, while extended endocrine therapy delays childbearing; HER2+ patients can typically undergo COS before neoadjuvant therapy, with pregnancy safety after trastuzumab washout (approximately 6 months) (Hong et al., 2023) [8]; TNBC’s aggressive biology and high chemotherapeutic intensity often favor ovarian tissue cryopreservation over time-intensive COS [8]; and BRCA1/2-associated cancers present unique challenges, including potentially diminished ovarian reserve, timing of risk-reducing salpingo-oophorectomy, and theoretical malignancy risks with ovarian tissue reimplantation. Despite this, existing FP guidelines remain largely uniform and do not provide subtype-specific guidance on uptake rates, stimulation outcomes, reproductive success, or oncologic safety—a critical knowledge gap this review addresses.

This review is, to our knowledge, the first systematic review to use tumor subtype stratification as the primary analytical framework, explicitly comparing FP outcomes across HR+, HER2+, TNBC, and BRCA-associated subtypes, and examining how subtype-specific biology and treatment context modulate FP outcomes. Prior systematic reviews have addressed FP safety or efficacy in breast cancer broadly without this subtype-stratified focus. Specifically, this review aims to: (1) compare FP methods across breast cancer subtypes; (2) identify subtype-specific challenges; and (3) propose pathways toward precision oncofertility care.

## 2. Materials and Methods

This systematic review was conducted following the Preferred Reporting Items for Systematic Reviews and Meta-Analyses (PRISMA) and the PRISMA-P guidelines for protocols [16].

### 2.1. Inclusion Criteria

Eligible studies included women with breast cancer who were younger than 40 years and/or explicitly described as premenopausal or adolescent and young adult. Breast cancer needed to be stratified by type, including ER+/PR+ (hormone receptor-positive), HER2-positive, triple-negative breast cancer (TNBC), or BRCA1/2 mutation carriers. The intervention of interest was any fertility preservation method, initiated before or during systemic therapy, including oocyte and/or embryo cryopreservation, ovarian tissue cryopreservation, GnRH agonists administered during chemotherapy, and controlled ovarian stimulation (COS), with or without protocol modifications. Eligible study designs included randomized controlled trials, cohort studies, and case–control studies.

### 2.2. Exclusion Criteria

Studies were excluded if (1) they included male patients or any animal or preclinical laboratory studies, (2) did not specify the subtype, or reported results from non-cancer populations, (3) did not report on fertility outcomes, (4) included patients with significant reproductive or endocrine comorbidities that independently affect ovarian reserve or fertility, such as PCOS, endometriosis, and uncontrolled diabetes. Ineligible study designs included case reports/series, reviews, editorials, conference abstracts without full-text articles, and opinion pieces, as well as studies older than 20 years and not published in English.

### 2.3. Outcomes

Primary fertility outcomes were post-treatment ovarian reserve measures (Anti-Müllerian hormone [AMH], follicle-stimulating hormone [FSH], and antral follicle count [AFC]) relative to baseline. Secondary outcomes included FP uptake and completion rates, ovarian stimulation response (e.g., oocyte/embryo yield, cycle cancellation), FP-related adverse events, fertility outcomes specifically in BRCA carriers, and, when available, pregnancy and live birth.

### 2.4. Search Strategy

A comprehensive literature search was conducted across 3 major databases, including Web of Science, Scopus, and PubMed, restricted to English-language studies published over the last 20 years (2004–2024). Search strategies were customized to the indexing system and syntax of each database. The search was restricted to peer-reviewed literature indexed in these three databases; gray literature, conference abstracts without full-text availability, and clinical trial registries were not separately searched, and this is acknowledged as a limitation (see Section 4.7). Manual reference list screening of all included full-text articles was performed as a supplementary step to identify any relevant studies not captured by the database search.

The following search formulas were applied:

PubMed: (“Breast Neoplasms”[MeSH] OR “breast cancer” OR “breast carcinoma”) AND (“Fertility Preservation”[MeSH] OR “fertility preservation” OR “oocyte cryopreservation” OR “embryo cryopreservation” OR “ovarian tissue cryopreservation” OR “GnRH agonist*” OR “gonadotropin-releasing hormone agonist*”) AND (“Pregnancy”[MeSH] OR “Live Birth”[MeSH] OR “Fertility”[MeSH] OR “ovarian reserve” OR “AMH” OR “Anti-Müllerian Hormone” OR “antral follicle count” OR “recurrence” OR “survival”) AND (“ER-positive” OR “PR-positive” OR “HER2-positive” OR “triple-negative” OR “TNBC”) AND (“young women” OR “premenopausal” OR “adolescent and young adult”[MeSH]).

Scopus: (TITLE-ABS-KEY (“fertility preservation” OR “oocyte cryopreservation” OR “embryo cryopreservation” OR “ovarian tissue cryopreservation” OR “GnRH agonists” OR “in vitro maturation”) AND TITLE-ABS-KEY (“breast cancer” OR “triple-negative” OR “HER2 positive” OR “BRCA mutation” OR “ER positive” OR “PR positive”) AND TITLE-ABS-KEY (young OR premenopausal OR “reproductive age”)) AND PUBYEAR > 1999 AND PUBYEAR < 2026 AND (LIMIT-TO (DOCTYPE, “ar”) OR LIMIT-TO (DOCTYPE, “re”)) AND (LIMIT-TO (LANGUAGE, “English”)).

Web of Science: ([mh “Breast Neoplasms”] OR “breast cancer” OR “breast carcinoma”) AND ([mh “Fertility Preservation”] OR “fertility preservation” OR “oocyte cryopreservation” OR “embryo cryopreservation” OR “ovarian tissue cryopreservation” OR “GnRH agonist*” OR “gonadotropin-releasing hormone agonist*”) AND ([mh “Pregnancy”] OR [mh “Live Birth”] OR “fertility” OR “ovarian reserve” OR “AMH” OR “Anti-Müllerian Hormone” OR “antral follicle count” OR “recurrence” OR “survival”) AND (“ER-positive” OR “PR-positive” OR “HER2-positive” OR “triple-negative” OR “TNBC”) AND (“young women” OR “premenopausal” OR “adolescent and young adult”).

### 2.5. Screening and Data Extraction

All records identified in the database searches were imported into Rayyan AI (Qatar Computing Research Institute, https://www.rayyan.ai, accessed on 7 June 2026), a reference management software, for initial organization and duplicate removal. A two-stage approach was followed: in the first stage, two independent reviewers screened the titles and abstracts of all records against the eligibility criteria. Records deemed potentially relevant by either reviewer were forwarded for full-text assessment. In the second stage, full-text articles were independently evaluated to ensure complete relevance. Any disagreements between the two primary reviewers at either stage were resolved through discussion, and if consensus could not be reached, a third reviewer would make the final decision. The search results were documented and summarized in a PRISMA flow diagram.

Data extraction was performed by two independent reviewers using a standardized extraction template to report details and variables needed for subtype-stratified analysis. It captured publication details, sample size, population characteristics, breast cancer subtype, fertility preservation intervention, primary and secondary fertility outcome measures, oncological outcome measures, and key findings. When data were missing or unreported, the reviewers noted ‘NR’ in the table.

### 2.6. Risk of Bias Assessment

Risk of bias assessment was conducted at the study level, independently by two reviewers, using validated tools tailored to specific study designs. Randomized controlled trials were assessed using the Cochrane Risk of Bias tool version 2 (RoB 2), which evaluates domains including the randomization process, deviations from intended interventions, missing outcome data, outcome measurement, and the selection of reported results. For non-randomized observational studies (cohort and case–control designs), they were assessed using the Risk Of Bias In Non-randomized Studies of Interventions tool version 1 (ROBINS-I), which evaluates bias in relation to a hypothetical target randomized trial across seven domains: bias due to confounding, selection of participants, classification of interventions, deviations from intended interventions, missing data, measurement of outcomes, and selection of the reported result. The assessment focused on the representativeness of breast cancer cohorts, the reliable and valid use of statistical controls, and the reliability of the outcome measures. The assessment results were used to interpret the strength of the evidence and incorporated into the final qualitative synthesis.

### 2.7. Data Synthesis

Given the expected clinical and methodological heterogeneity across studies, we planned a primarily narrative synthesis. Formal meta-analysis was not performed because the conditions required for valid quantitative pooling were not met: (1) clinical heterogeneity—stimulation protocols, patient age, chemotherapy regimens, and endocrine therapy varied substantially; (2) methodological heterogeneity—14 of 19 studies were retrospective cohorts with differing designs and comparison groups; and (3) outcome heterogeneity—ovarian reserve was measured using non-standardized combinations of AMH, FSH, and AFC at varying post-treatment timepoints, and live birth rates were reported in only a minority of studies. These same factors precluded quantitative subgroup analyses by breast cancer subtype; fewer than three studies per subtype reported comparable outcomes amenable to pooling. All evidence synthesis is therefore narrative.

Studies were grouped by FP modality (COS with or without letrozole/tamoxifen; GnRH agonists; IVM/OTC) and, where data allowed, by tumor subtype. Subgroup analyses by tumor subtype and FP modality were planned but were ultimately not feasible due to the heterogeneity described above. Each included study represented a unique, non-overlapping patient cohort; no instances of overlapping populations from the same institution were identified.

Given the absence of prespecified quantitative thresholds applicable to all included study designs, subtype-specific effectiveness was assessed narratively across three domains: ovarian stimulation response, post-treatment ovarian reserve, and, where reported, live birth rates. Confidence in subtype-specific conclusions was graded qualitatively based on the number, consistency, and methodological quality of contributing studies. Evidence was considered more robust for HR+ patients (the largest number of consistent studies) and more limited for TNBC, HER2+, and BRCA1-specific subgroups.

## 3. Results

### 3.1. Study Selection

**Figure 1:**
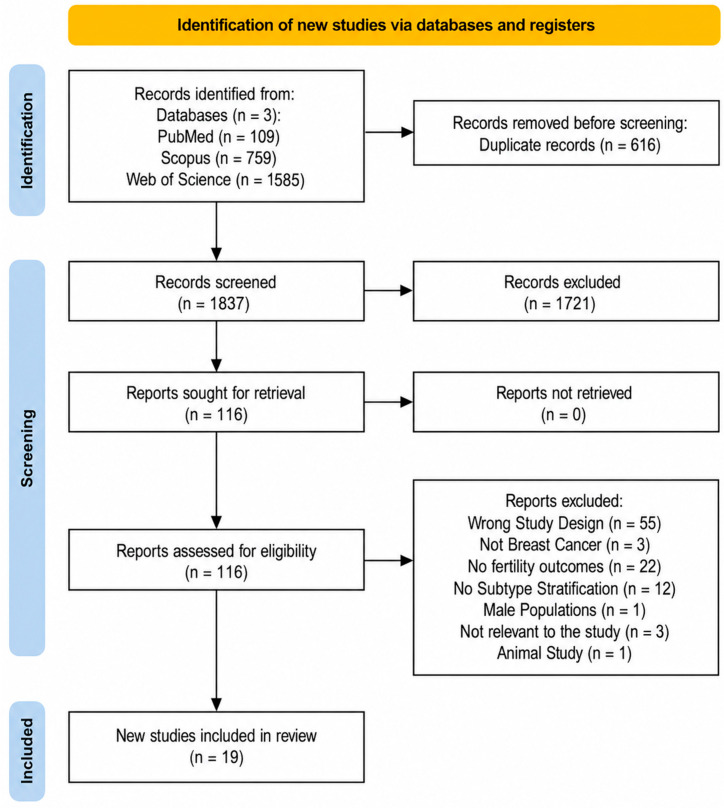
PRISMA flowchart.

A total of 2453 records were initially identified: PubMed (*n* = 109), Scopus (*n* = 759), and Web of Science (*n* = 1585). After removing 616 duplicates, 1837 records were screened. Of these, 1721 were excluded. From the remaining 116 articles assessed in full text, 97 were excluded (wrong design *n* = 55, no fertility outcomes *n* = 22, no subtype stratification *n* = 12, non-breast cancer *n* = 3, irrelevant *n* = 3, male-only *n* = 1, animal study *n* = 1). Ultimately, 19 studies met inclusion criteria (Figure 1).

### 3.2. Study Characteristics

The final studies included 2 randomized controlled trials and 17 cohort studies from Europe (*n* = 8), North America (*n* = 6), Asia (*n* = 3), and multicenter/international cohorts (*n* = 2). Most were retrospective (*n* = 14) or prospective (*n* = 3) cohorts. Interventions comprised COS with letrozole/tamoxifen-modified protocols (*n* = 12), GnRH agonists during chemotherapy (*n* = 4), and IVM/OTC (*n* = 3). Thirteen of 19 studies stratified outcomes by tumor biology or treatment context; this subtype-stratified evidence base is the critical focus of this review. HR+ and BRCA-associated disease were the best-represented subtypes, while TNBC and HER2+ were underrepresented, with fewer than five studies reporting subtype-specific outcomes for these groups—representing a key evidence gap. Premenopausal status was confirmed by self-report, menstrual history, or hormonal assessment (FSH/estradiol) in most studies; some cohorts included women up to age 44–45 who may have had limited ovarian reserve, introducing potential heterogeneity in baseline fertility status. All studies evaluated fertility preservation strategies in premenopausal women diagnosed with breast cancer. The characteristics of all included studies are summarized in Table 1.

### 3.3. Bias Assessment

**Figure 2:**
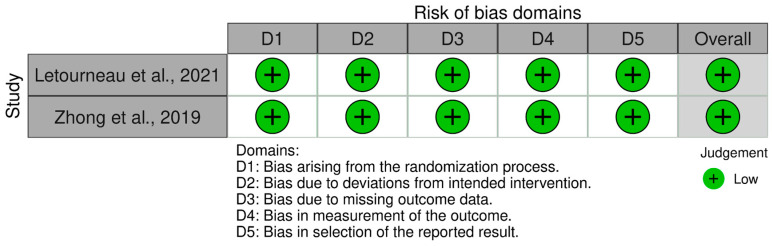
Risk-of-bias assessment using the Cochrane RoB 2.0 tool for RCTs. [22,32].

**Figure 3:**
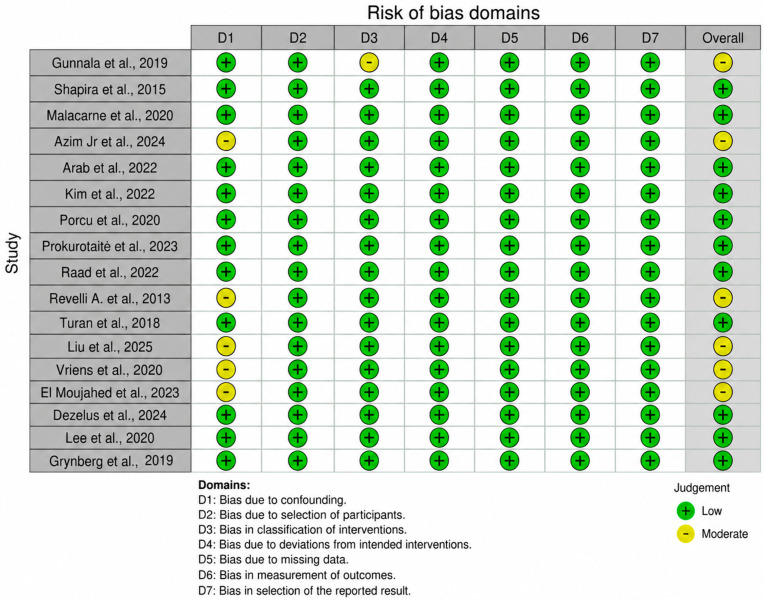
Risk-of-bias assessment using the ROBINS-I tool for cohort studies [17,18,19,20,21,23,24,25,26,27,28,29,30,31,33,34,35].

Among the 2 randomized controlled trials, both demonstrated low risk of bias across all Cochrane RoB 2 domains. Among the 17 non-randomized studies assessed with ROBINS-I, 11 received an overall low risk of bias rating, and 6 received a moderate rating [20,23,25,27,29,30], primarily in Domain 1 (bias due to confounding), reflecting insufficiently controlled variation in age, baseline ovarian reserve, disease stage, and treatment intensity. These figures are summarized in Figure 2 and Figure 3.

It should be noted that moderate confounding in observational cohorts (for example, healthier patients with better baseline ovarian reserve being preferentially selected for GnRH agonist co-administration) may bias associations toward apparent benefit for ovarian protection endpoints. This interpretive point is discussed further in Section 4.1.

### 3.4. Summary of Evidence

In HR+ patients undergoing COS with letrozole or tamoxifen (the subgroup with the most consistent evidence) FP was feasible and produced acceptable ovarian stimulation responses. Evidence of feasibility was weaker for TNBC and HER2+ patients, where fewer subtype-specific data are available. Notably, controlled ovarian stimulation protocols using letrozole or tamoxifen were consistently associated with lower estradiol exposure, with most data relating to ovarian response endpoints; robust data on live births and long-term oncologic outcomes following modified COS remain limited. Among BRCA mutation carriers, several studies found outcomes comparable to non-carriers, although results are inconsistent between studies and between BRCA1 and BRCA2 carriers, with small sample sizes limiting firm subtype-specific conclusions. Some studies, particularly [21], reported lower mature oocyte yields and AMH levels in BRCA1-positive patients specifically.

Studies assessing GnRH agonists during chemotherapy suggested a protective effect on ovarian function, with lower ovarian failure rates and better post-treatment menstrual or AMH recovery. However, GnRH agonist use should be understood as ovarian function preservation (reducing chemotherapy-induced ovarian failure) rather than as an equivalent to oocyte or embryo cryopreservation, which directly preserves gametes. Pregnancy and live birth data for GnRHa alone remain limited across the included studies.

In terms of long-term follow-up, data across included studies with medium-term oncologic follow-up (predominantly 3–5.5 years) showed no signal of increased recurrence or mortality attributable to FP. This should be interpreted as reassuring medium-term safety, not a definitive oncologic guarantee, given that most studies were underpowered for rare or late events and follow-up beyond 5 years is sparse. Overall, the evidence suggests that FP is feasible across breast cancer subtypes, but conclusions regarding comparative effectiveness and long-term reproductive outcomes remain constrained, particularly for TNBC, HER2+, and BRCA1-specific subgroups.

## 4. Discussion

This systematic review synthesized the findings of 19 studies examining fertility preservation outcomes in women with breast cancer. The findings suggest that FP is procedurally feasible in young women with breast cancer across most subtypes, with ovarian stimulation responses generally within acceptable ranges in the subgroups where evidence is most robust—primarily HR+ patients. These conclusions must be interpreted within the context of a predominantly retrospective evidence base, heterogeneous outcome definitions, and follow-up periods that are insufficient to exclude late oncologic risks or to characterize long-term reproductive success. Substantial heterogeneity in study design, patient populations, tumor characteristics, and outcome reporting limited direct comparison across studies and precluded quantitative pooling.

### 4.1. Ovarian Function Preservation

A key method investigated was ovarian function preservation through the use of gonadotropin-releasing hormone (GnRH) agonists during chemotherapy. Overall, GnRHa use in the included studies was associated with a reduction in chemotherapy-induced ovarian failure and improved ovarian recovery [19,31,32]. For example, a randomized controlled trial (*n* = 98) reported that the rate of one-year ovarian failure was significantly reduced from 80.6% in the chemotherapy-alone group to 44.7% in the GnRHa group (*p* = 0.002), with benefit observed across HR+ and HR− subgroups, though statistical significance was reached only in the HR+ subgroup (*p* = 0.029); the HR− subgroup showed a numerical trend that did not reach significance (*p* = 0.111) [32]. Likewise, another observational study reported the resumption of menses in 97% of patients and anti-Müllerian hormone levels greater than 1 ng/mL in 70% at 12 months [31]. Across studies, younger age and higher baseline ovarian reserve were associated with improved outcomes, whereas exposure to alkylating chemotherapy was consistently linked to increased gonadotoxicity [31,33,35]. Notably, the effect of tumor subtype on ovarian recovery was less consistent across investigations [27].

Importantly, GnRH agonist co-administration during chemotherapy should be understood as ovarian function preservation—reducing the rate of chemotherapy-induced ovarian failure and supporting menstrual recovery—rather than as an equivalent to oocyte or embryo cryopreservation, which directly preserve gametes for future use. This distinction is clinically important: women who receive only GnRHa during chemotherapy retain no cryopreserved material and rely entirely on spontaneous ovarian recovery to conceive. Pregnancy and live birth data specifically attributable to GnRHa use (as opposed to spontaneous recovery) remain limited in the included studies. The interpretive caution noted in Section 3.3 regarding confounding also applies here: healthier patients with better baseline reserve may be preferentially selected for GnRHa, potentially inflating apparent effectiveness.

### 4.2. Controlled Ovarian Stimulation and Cryopreservation Outcomes

Further, the majority of included studies evaluated controlled ovarian stimulation for oocyte or embryo cryopreservation. These studies consistently confirmed that fertility preservation is feasible prior to chemotherapy, with high procedural success rates. A procedure success rate of 89.5% has been reported, with a mean retrieval of 12.8 oocytes and 9.8 mature oocytes vitrified [17]. Results on oocyte yield with modified protocols are not entirely consistent. Most studies, including two randomized controlled trials [17,22], found no statistically significant difference in mature oocyte yield between letrozole- or tamoxifen-modified COS and standard gonadotropin stimulation. However, Revelli A et al. [23] reported 1–2 fewer oocytes per cycle with letrozole-gonadotropin in HR+ patients (6.6 ± 3.5 vs. 8.0 ± 5.0 oocytes available for cryostorage; *p* = 0.038) compared with gonadotropin alone in ER-negative patients, suggesting a possible modest trade-off in yield. This comparison involved different patient subgroups and was not a protocol-matched randomized analysis; the observed difference should therefore be interpreted cautiously. These protocols are associated with markedly lower peak estradiol levels, supporting their use in HR+ disease. The clinical significance of any yield reduction and its impact on cumulative live birth probability remain unstudied. Furthermore, most data relate to ovarian response endpoints; evidence on live births and long-term oncologic outcomes following modified COS is limited, and conclusions about true effectiveness for childbearing must be tempered accordingly.

The oncologic safety of COS in HR+ patients is supported by several lines of evidence. Vriens et al. reported no compromise of oncologic outcomes at median 52-month follow-up in a prospective cohort that included a majority of HR+ patients [29]. Azim Jr et al., in a secondary analysis of the POSITIVE trial (*n* = 516, all HR+), found no significant increase in 3-year recurrence risk associated with prior ovarian stimulation at diagnosis [30]. Dezellus et al. reported 5-year disease-free survival of 82% and overall survival of 90% in their tamoxifen-COS cohort [17]. The mechanistic basis for safety is supported by letrozole or tamoxifen co-administration during stimulation, which suppresses peak estradiol, mitigating the theoretical concern of estrogen exposure in ER+ disease. These data are reassuring but should be interpreted as medium-term safety signals rather than definitive long-term guarantees.

### 4.3. Impact of BRCA Mutation Status

In terms of genetic risk, the effect of BRCA mutation status on fertility preservation outcomes was evaluated across 11 studies, with generally consistent findings showing that ovarian reserve and stimulation response were comparable between mutation carriers and non-carriers [19,24,26,34]. However, results are inconsistent across studies, and a possible differential impact for BRCA1 vs. BRCA2 carriers cannot be resolved with current data. Some studies reported lower mature oocyte yield and reduced maturation rates among BRCA1 mutation carriers specifically [21,25], while others found no significant differences [20,25]. Moujahed et al. [25]—the largest study assessing ovarian response to COS in BRCA carriers—found no statistically significant difference in ovarian reserve markers or response between BRCA1 and BRCA2 subgroups, consistent with Gunnala et al. [20]. In contrast, Porcu et al. [21] reported lower serum AMH and fewer mature oocytes specifically in BRCA1-positive patients. In another study, lower maturation rates (78.6% vs. 85.7%) and fewer mature oocytes were observed in patients with BRCA mutations [25] (El Moujahed et al., 2023). However, some studies reported lower mature oocyte yield and reduced maturation rates among BRCA1 mutation carriers [18,21,25,28]. Overall, these results suggest that while BRCA1 mutations may be associated with measurable deficits in ovarian reserve and oocyte quality, it remains unclear whether these factors significantly reduce the clinical success rates of fertility preservation procedures. The mechanistic basis for potential BRCA1-specific impairment merits elaboration. BRCA1 and BRCA2 proteins play essential roles in homologous recombination repair of double-strand DNA breaks. During oocyte meiosis (particularly prophase I), DNA strand breaks are physiologically generated and require efficient repair; defective homologous recombination in BRCA-mutant oocytes has been hypothesized to trigger accelerated apoptosis and primordial follicle depletion [25] (Moujahed et al., 2023). Consistent with this, a meta-analytic dataset cited by Moujahed et al. suggests AMH levels are approximately 1.0 ng/mL lower in BRCA-mutated patients undergoing FP for breast cancer compared to non-carriers [25]. Additionally, BRCA1 carriers more frequently present with TNBC and high-grade tumors, and tumor aggressivity itself may independently impair ovarian response—making it difficult to disentangle the respective contributions of germline mutation and tumor biology to observed yield differences.

These findings should be interpreted as ‘no large, clearly established impairment of overall FP effectiveness attributable to BRCA mutation status’ rather than confirmation of fully preserved effectiveness. BRCA1 mutation carriers may warrant individualized counseling about potential variability in oocyte maturity and ovarian response, and earlier or repeated FP attempts may be advisable in selected cases.

### 4.4. In Vitro Maturation, In Vitro Fertilization, and Ovarian Tissue Cryopreservation

Turning attention to alternative techniques, such as in vitro fertilization, in vitro maturation, and ovarian tissue cryopreservation, IVM was identified as an alternative fertility preservation strategy, particularly for patients requiring urgent initiation of chemotherapy and therefore unable to undergo conventional ovarian stimulation. Among these approaches, Raad et al. reported a maturation rate of approximately 58%, with a mean of 5.8 mature oocytes vitrified per cycle, indicating lower efficiency than standard protocols [35].

Despite its feasibility, these cohorts were impacted by both patient and disease-related factors. Specifically, reduced ovarian reserve, as indicated by anti-Müllerian hormone levels below 1.5 ng/mL and a low antral follicle count, was associated with lower maturation rates [31,32,35]. Additionally, Raad et al. suggested that more aggressive tumor subtypes, including HER2-positive disease, high tumor grade, and triple-negative breast cancer, were linked to reduced success.

IVM and OTC should be presented to patients as approaches that remain relatively experimental in the breast cancer setting, with lower established oocyte competence than conventional COS and with essentially no live birth outcome data available in this specific population. IVM is best considered in combination with OTC to maximize cumulative fertility potential, though this combined approach also lacks long-term efficacy data and should be framed accordingly in patient counseling.

The observation by Raad et al. that more aggressive tumor subtypes (including TNBC, high-grade disease, and HER2-positive) may be associated with reduced IVM success is hypothesis-generating and derived from a single retrospective study with small subgroup numbers [35]. It should not be interpreted as a causal relationship or a contraindication to IVM in these subtypes, but rather as a signal warranting prospective investigation.

### 4.5. Post-Treatment Fertility Outcomes

Turning now to post-treatment fertility outcomes, reporting was sparse: only 6 studies provided long-term follow-up data, representing less than 20% of the overall cohorts. Among these, assisted reproductive outcomes were generally favorable. In a large cohort of hormone receptor-positive patients (*n* = 518), the use of cryopreserved embryos was associated with a significant increase in pregnancy rates, with embryo transfer doubling the odds of conception (odds ratio 2.41) [30]. Vriens et al. reported a 5-year live birth rate of 27% (95% CI 17–38%) and a 5-year ovarian function recovery rate of 92% in their prospective cohort [29].

Live birth outcomes following controlled ovarian stimulation were also encouraging, with reported rates comparable between BRCA mutation carriers and non-carriers [35]. Nonetheless, utilization of cryopreserved material remained low, with return-to-use rates ranging from 5% to 10%. Notably, spontaneous pregnancies were also reported, occurring in approximately 22.1% of patients at five years in one cohort utilizing tamoxifen-based stimulation protocols [17].

Regarding treatment delay, in the cohort reported by Zhong et al. [32], FP was associated with a chemotherapy initiation delay of less than one week; this finding should be interpreted within the context of that specific study design rather than as a universal estimate applicable to all FP modalities. Regarding oncologic safety, no signal of increased recurrence was detected in any included study over the available follow-up periods (predominantly 3–5.5 years); this should be interpreted as ‘no detectable signal in medium-term observational and trial data’ rather than a definitive safety guarantee, given that most studies were underpowered for rare or late oncologic events. No increase in recurrence was observed after COS/FP (including HR+ cases) [29,30].

Overall, no clear superiority of one fertility preservation modality over another could be established, largely due to heterogeneity in follow-up duration, utilization of assisted reproductive technology, and reporting of partner status. Furthermore, key reproductive outcomes such as time to pregnancy and cumulative pregnancy rates were infrequently reported, further challenging comparison between modalities.

### 4.6. Clinical Implications and Recommendations for Practice

These findings support the early, routine integration of oncofertility counseling into the multidisciplinary breast cancer care pathway for all premenopausal women. Fertility preservation (FP) should be discussed before systemic therapy begins, as evidence consistently indicates that FP does not substantially delay cancer treatment and is not associated with increased oncologic recurrence or mortality, even in aggressive subtypes. Based on currently available medium-term data, FP has not been associated with a statistically significant increase in recurrence or mortality in the included studies; this conclusion is most applicable to HR+ and BRCA-associated populations with 3–5 years of follow-up, and evidence for TNBC and HER2+ subtypes remains more limited. Tumor subtype should additionally inform counseling on expected outcomes: women with triple-negative breast cancer and high-grade disease may have lower baseline ovarian reserve and yield fewer oocytes regardless of the stimulation method employed, and this information should be used to set realistic expectations and prioritize earlier referral [35]. For HR+ patients, letrozole-based controlled ovarian stimulation (COS) is a preferred protocol to lessen potential risks associated with peak estradiol exposure [36].

Where letrozole is unavailable or not tolerated, tamoxifen-gonadotropin represents a well-supported alternative, having demonstrated equivalent mature oocyte yields in a randomized controlled trial and a large prospective cohort [17,22]. The adoption of random-start GnRH antagonist protocols is further recommended in practice, as oocyte yield does not differ significantly by menstrual cycle phase at stimulation onset [18,26]. Furthermore, BRCA1 mutation carriers may warrant individualized counseling regarding potential variations in oocyte maturity and ovarian response, and earlier or repeated FP attempts may be advisable in selected cases [21,37]. When COS is contraindicated or chemotherapy is critically urgent, in vitro maturation (IVM) of immature oocytes represents a viable alternative initiable within days of diagnosis; given the lower competence of IVM-derived oocytes relative to those from conventional COS, IVM is best considered in combination with ovarian tissue cryopreservation (OTC) to maximize cumulative fertility potential [34,35]. In settings where established FP services are unavailable, administration of a gonadotropin-releasing hormone agonist (GnRHa) may be considered to preserve ovarian function, particularly in hormone receptor-negative disease, though it should be clearly distinguished from gamete or embryo cryopreservation [38].

### 4.7. Limitations and Methodological Considerations

This review has several methodological limitations that should be considered when interpreting its findings. Regarding the search strategy, restricting the search to three databases and English-language publications may have introduced selection and language bias, potentially omitting relevant studies indexed in regional databases or published in other languages, including those from regions where breast cancer is highly prevalent. The exclusion of studies older than 20 years, while justified by the rapid evolution of FP techniques and treatment protocols, may have excluded early cohort data relevant to long-term reproductive outcomes. This review was not prospectively registered.

At the study level, 14 of 19 included studies were retrospective in design, which carries an inherent risk of selection bias and unmeasured confounding. Women who pursued FP may differ systematically from those who did not in disease severity, socioeconomic status, and personal preferences, thereby limiting generalizability. Although RCTs demonstrated low risk of bias on the Cochrane RoB tool, cohort and non-randomized studies showed moderate risk of bias in domains related to confounding and intervention protocol variability. Outcome definitions were also heterogeneous across studies: ovarian reserve was assessed using AFC, AMH, and FSH at non-standardized time points, and the criteria used to define successful preservation of ovarian function varied substantially. This heterogeneity precluded formal meta-analysis and limits the precision of the review’s conclusions.

Finally, long-term reproductive outcomes—including pregnancy rates, cumulative ART success, and live birth rates—were reported in fewer than one-third of included studies, and return-to-use rates for cryopreserved material were low across the literature. The predominance of surrogate ovarian endpoints (AMH, oocyte yield) rather than true fertility endpoints (live births) is a critical limitation that must temper conclusions about FP ‘effectiveness’ throughout this review. These gaps collectively reduce the certainty of the synthesized evidence and limit the ability to draw firm conclusions about the comparative effectiveness of different FP strategies across patient subgroups.

Addressing inconsistent outcome reporting is a research imperative. We propose adoption of a minimum core outcome set for future oncofertility research, encompassing at minimum: number of mature oocytes retrieved, live birth rate, time to pregnancy, post-treatment AMH and menstrual recovery, and cancer-free survival. Adherence to CONSORT and STROBE reporting standards, with mandatory subgroup reporting by tumor subtype and the establishment of international prospective registries with standardized data collection, are also recommended.

### 4.8. Directions for Future Research

Future research should prioritize prospective, multicenter, subtype-stratified trials powered for live birth as a primary endpoint. Establishing robust evidence on live birth rates, cumulative ART success, and late-term oncologic safety is essential. Furthermore, comparative effectiveness research is required to define optimal FP strategies, such as COS versus ovarian tissue cryopreservation, across specific molecular subtypes. Additionally, research should examine the biological mechanisms underlying reduced oocyte maturation in BRCA1 carriers. Researchers suggested that BRCA1 has a role in meiotic spindle assembly and DNA damage response in oocytes, and that mutation carriers have significantly lower primordial follicle densities and higher rates of DNA double-strand breaks in oocytes [39,40]. These findings can inform strategies to improve gamete quality.

A critical and urgent research gap concerns the reproductive safety of newer systemic therapies. CDK4/6 inhibitors target cell-cycle pathways vital for follicular development, raising theoretical concerns about ovarian toxicity that remain unconfirmed by current clinical data [41]. PARP inhibitors have been shown to cause primordial follicle depletion in preclinical models, but human data are virtually absent. Immune checkpoint inhibitors, such as pembrolizumab, have unknown implications for ovarian reserve. Definitive reassurance for these agents is currently impossible; prospective evaluation of ovarian reserve markers, menstrual function, and fertility outcomes in women exposed to these therapies is urgently required, and FP should ideally be completed before their initiation.

Biomarker-guided FP strategies integrating AMH, AFC, and tumor biology to predict stimulation response and individualize protocols represent an important future direction, as does AI-assisted reproductive risk prediction incorporating tumor and treatment characteristics. Additionally, equity-focused research is imperative, as race, ethnicity, socioeconomic status, insurance coverage, and geography significantly limit equitable access to FP. Future studies must include underrepresented populations, pre-specified equity analyses, and prospective testing of structural interventions, including patient navigation and financial support.

## 5. Conclusions

This systematic review demonstrates that fertility preservation is procedurally feasible and associated with acceptable ovarian stimulation responses in young women with breast cancer when initiated before systemic therapy, with the strongest evidence in HR+ patients. Letrozole- and tamoxifen-modified COS protocols were associated with acceptable oocyte yields while limiting estradiol exposure. GnRH agonist co-administration during chemotherapy was associated with reduced rates of ovarian failure and improved recovery of ovarian function (most consistently in hormone receptor-negative disease), though it should be understood as ovarian function preservation rather than as an equivalent to gamete cryopreservation. No included study identified a statistically significant increase in cancer recurrence or mortality attributable to FP; however, this reassurance is explicitly limited by predominantly retrospective designs, follow-up durations of 3–5.5 years, and studies that were underpowered to detect rare or late oncologic events.

Patient and tumor characteristics (including BRCA mutation status, tumor grade, and hormone receptor profile) influence stimulation outcomes to varying degrees and should inform individualized counseling rather than serve as grounds to withhold FP. Conclusions regarding BRCA1-specific outcomes, TNBC, HER2+, and IVM/OTC remain constrained by small sample sizes, inconsistent results, and the lack of live-birth data. These conclusions are tempered by the predominance of retrospective designs, heterogeneity in outcome definitions, and a paucity of long-term reproductive follow-up data. Future prospective, subtype-stratified studies with standardized outcomes (in particular live birth rates) and longer follow-up are urgently needed. Until such evidence is available, early, multidisciplinary oncofertility counseling remains the cornerstone of care for premenopausal women with breast cancer.

## Figures and Tables

**Table 1:** Characteristics and Fertility Preservation Outcomes of Included Studies.

Study Info	Study Design	Sample Size	Population Characteristics + BC Subtype	Cancer Treatment	FP Timing	FP Method	Fertility Outcomes	Follow Up	Key Findings
**Controlled Ovarian Stimulation (COS) Studies**									
Dezellus et al., 2024 [17]	Multicentric prospective cohort study	95	Most patients had grade II–III carcinoma (94.6 %) and Estrogen Receptor (ER) positive cancer (67 %)	69 pts (72.6%) received FEC → Taxane (3 FEC + 3 taxane); 36 NAC (37.9%), 59 adjuvant (62.1%).	• Neoadjuvant Group: Median time from first oncological visit to chemotherapy initiation: 3.6 weeks • Adjuvant Group: Median time from surgery to chemotherapy initiation: 6.0 weeks	Tamoxifen-controlled ovarian hyperstimulation (COS) for oocyte/embryo cryopreservation	Procedure success 89.5%; 10.5% canceled (mostly poor response); mean oocytes collected 12.8, mature vitrified 9.8; stimulation 10.4 days; yield similar across cycle start phases.	• Median Follow-up: 5.5 years (95% CI: 5.4–5.7) • Loss to Follow-up: 2 patients	Tamoxifen-COS effective for FP (89.5% success, 9.8 oocytes); outcomes similar to letrozole-COS; no significant chemo delay; 5-yr DFS 82%, OS 90%; flexible start phase; 22.1% spontaneous pregnancy low oocyte use return rate (5.3%).
Kim et al., 2022 [18]	Retrospective study	117	TNBC, ER, PR, HER2-+, BRCA1, BRCA2, and both BRCA1 and BRCA2	Not applicable; FP performed before gonadotoxic therapy.	Performed before gonadotoxic therapy.	Oocyte/embryo cryopreservation	BRCA carriers: similar ovarian reserve, lower oocyte yield; mature oocyte rate and 5-yr pregnancy rate comparable.	Not Reported	BRCA carriers had similar ovarian reserve but lower oocyte yield; TNBC patients had higher mature oocyte rates.
Shapira et al., 2015 [19]	Retrospective matched cohort study	62	Of the included carriers, 43 had a mutation in the BRCA1 gene 17 had a mutation in the BRCA2 gene, and 1 had mutations in both genes. In 1 additional carrier, definite information regarding the involved mutated gene (BRCA1 or BRCA2) was not available.	Not Reported.	Breast Cancer Patients underwent IVF for fertility preservation before chemotherapy	FP used COH for IVF: 54.8% long GnRH agonist, remainder GnRH antagonist; 19 HR+ patients received tamoxifen 20 mg/day during stimulation.	No Significant Differences Between BRCA+ and BRCA Groups	Retrospective matched cohort study	BRCA mutation carriers demonstrate normal ovarian response and comparable IVF outcomes (oocyte yield, fertilization, and pregnancy rates) relative to non-carriers. These findings support the use of standard IVF protocols and indicate that BRCA status should not alter fertility preservation recommendations.
Gunnala et al., 2019 [20]	Retrospective cohort study	795	Total: 329 BC patients (52 BRCA+, 277 BRCA−).	Not detailed; FP performed before gonadotoxic therapy	Performed before gonadotoxic therapy.	Oocyte cryopreservation after COS (mostly antagonist protocol) + letrozole	Findings: BRCA carriers had higher AFC (15.5 vs. 12.6) and more mature oocytes cryopreserved (14.0 vs. 10.4).	Not reported (single-cycle, pre-treatment).	BRCA carriers have similar reproductive potential at baseline to noncarriers.
Porcu et al., 2020 [21]	Prospective cohort study	46	Young women with breast cancer, with and without BRCA mutations. Mean age ~32.5 years (20–40), premenopausal. Stage I–II.	All referred for FP before starting chemo/radiotherapy.	Before starting chemotherapy and radiotherapy.	.COS with GnRH agonist protocol + r-FSH, + letrozole for hormone-sensitive tumors.	Findings: BRCA1+ had lower AMH and yielded fewer mature oocytes vs. BRCA− BC and controls. BRCA2+ did not differ significantly.	Not applicable to this study.	Total oocytes were similar, but BRCA carriers had lower maturation and fewer mature oocytes; earlier or repeated FP may benefit BRCA-positive women.
Letourneau et al., 2021 [22]	Open-label, single-institution n, randomized controlled trial	134	Premenopausal women (18–44) with newly diagnosed non-metastatic breast cancer; randomized groups had ER+ disease, comparison group had ER– disease.	Patients were newly diagnosed and had not yet begun chemotherapy at the time of FP.	Performed after diagnosis but before the start of chemotherapy.	Oocyte or embryo cryopreservation using random-start GnRH antagonist protocol with gonadotropins; Those with estrogen-receptor-positive (ER+) breast cancer were randomized to tamoxifen-gonadotropin or letrozole-gonadotropin; randomized groups received tamoxifen (20 mg/day) or letrozole (5–10 mg/day).	Mature oocyte yield: Tamoxifen-Gn 12.0, Letrozole-Gn 11.6, Gn-only 12.4; no significant differences (*p* = 0.81). Peak estradiol was lower in the letrozole group (642 pg/mL) by design.	Follow-up was only for the duration of the ovarian stimulation cycle. No long-term follow-up for cancer or obstetrical outcomes is reported in this paper.	Tamoxifen-gonadotropin and letrozole-gonadotropin produced a similar number of mature oocytes. Women who received either tamoxifen-gonadotropin or letrozole-gonadotropin had a similar number of oocytes to the gonadotropin-only group.
Revelli et al., 2013 [23]	Multicenter Retrospective cohort study	75	50 ER+ breast cancer patients undergoing COH to cryopreserve oocytes before gonadotoxic chemotherapy with a Letrozole plus gonadotropins (Le+Gn) protocol were compared with those of 25 young women with ER- breast cancer, submitted to COH using a protocol with gonadotropins alone (Gn-only).	Patients were scheduled for gonadotoxic chemotherapy after oocyte retrieval.	Performed before gonadotoxic therapy.	Oocyte cryopreservation (slow freezing or vitrification) following controlled ovarian stimulation	The Le+Gn protocol implied a significantly lower total Gn consumption and allowed to maintain significantly lower circulating E2 levels at all checkpoints throughout stimulation the Le+Gn protocol allowed a significantly lower yield of oocytes available for cryostorage	No follow-up reported. The study focused on outcomes of the ovarian stimulation cycle itself.	Letrozole with gonadotropins yields fewer mature oocytes than gonadotropins alone but significantly lowers estradiol, highlighting a trade-off between fertility preservation and cancer safety.
Prokurotaite et al., 2023 [24]	Retrospective single-center cohort study.	85	85 patients were included in the 3 groups: (1) patients diagnosed with BC without a gBRCA PV, (2) patients diagnosed with BC with a gBRCA PV, and (3) healthy gBRCA PV carriers who underwent FP or PGT-M cycles	Not applicable: Fertility preservation occurred before starting any cancer treatment. Nearly all breast cancer patients were planned for (neo)adjuvant chemotherapy; anti-HER2 therapy was more frequent in non-BRCA carriers.	Performed before gonadotoxic therapy.	Ovarian stimulation for fertility preservation.	Fertility outcomes were similar between BRCA-positive and BRCA-negative patients, with comparable total oocytes retrieved (5–6 mature oocytes) and consistently high maturation rates (>80%).	Fertility follow up is limited as few patients.	Neither BC nor gBRCA PV significantly affects ovarian reserve and FP efficacy in terms of the number of mature oocytes retrieved.
El Moujahed et al., 2023 [25]	Retrospective cohort study	311	Inclusion criteria were a histologically confirmed diagnosis of BC, known BRCA status, and having undergone COH in the context of emergency FP with oncologists’ authorization	Not applicable: Fertility preservation occurred before starting any cancer treatment.	Performed urgently before gonadotoxic therapy.	Ovarian stimulation for fertility preservation.	The mean number of oocytes recovered, as well as the FORT The index did not significantly differ between the BRCA-mutated and non-mutated groups; Oocyte maturation rates were significantly altered in the BRCA-mutated group in comparison to the non-mutated group, leading to a lower number of MII oocytes	Not applicable to this study.	a BRCA pathogenic variant does not affect the response to COH in terms of number of oocytes retrieved, FORT, or oocyte retrieval rates but may alter the capacity of oocytes to reach the MII stage
Malacarne et al., 2020 [26]	Retrospective observational study	61	Breast cancer patients were categorized into multiple predefined groups: High-grade (n = 23) vs. low-grade (n = 24), High-stage (n = 14) vs. low-stage (n = 33), ER-positive (n = 36) vs. ER-negative (n = 11), TNBC (n = 6) vs. non-TNBC	All patients were scheduled to begin (neo)adjuvant chemotherapy after FP.	Performed before gonadotoxic therapy.	Ovarian stimulation for fertility preservation. using GnRH antagonist or PPOS protocols with recombinant FSH, hMG, or r-FSH + r-LH;	Breast cancer prognostic factors did not impact ovarian stimulation response or fertility preservation outcomes. The numbers of mature oocytes and total oocytes, and the estradiol response, were comparable across all prognostic groups.	Not applicable.	Authors conclude that these cancer characteristics should not be considered limiting factors when counseling patients about fertility preservation efficacy.
Liu et al., 2025 [27]	Retrospective cohort study	147	In the analysis of hormone receptor profiles, women were categorized according to their ER, PR, and HER-2 status. Patients who tested negative for ER, PR, and HER-2 were categorized as TNBC. The patients were subsequently divided into two groups: (1) individuals characterized as ER-positive vs. those classified as ER-negative, and (2) those with TNBC vs. non-TNBC.	Not applicable: All participants were newly diagnosed and underwent fertility preservation before beginning any cancer chemotherapy.	Performed before gonadotoxic therapy.	All patients underwent either oocyte or embryo cryopreservation with the administration of either gonadotropin-releasing hormone (GnRH) antagonist or progestin-primed ovarian stimulation (PPOS) protocols.	No significant differences in mature or total oocytes retrieved or peak estradiol were seen across subgroups (grade, stage, ER status, TNBC); patients exhibiting ER positivity demonstrated a comparable number of mature oocytes, collected oocytes, and peak estradiol levels; Patients with TNBC also exhibited a similar number of collected mature oocytes, total oocytes, and peak estradiol levels compared to patients without TNBC	Short-term oncologic follow-up.	BRCA mutation status does not significantly impair ovarian response during fertility preservation; the study showed similar ovarian stimulation response and fertility preservation outcomes among breast cancer patients with different prognostic factors.
Turan et al., 2018 [28]	secondary analysis of a prospective database	145	In the LF group, 21 had BRCA mutations; breast cancer patients undergoing controlled ovarian stimulation for fertility preservation. Subgroup analysis included BRCA mutation carriers and non-carriers.	All participants were newly diagnosed and underwent fertility preservation before beginning any cancer therapy.	Performed before gonadotoxic therapy.	Ovarian stimulation for fertility preservation.	BRCA mutations produced fewer oocytes and embryos compared to those who were BRCA negative or untested; After adjusting for age and BMI, these differences became more prominent with marginally lower fertilization rates in women with BRCA mutations	Short-term oncologic follow-up (2–3 years)	Letrozole-supplemented ovarian stimulation is effective and safe. BRCA mutation status does not significantly impair ovarian response during fertility preservation.
Vriens et al., 2020 [29]	Prospective cohort study with long-term follow-up.	118	Young women with early-stage breast cancer.	(Neo)adjuvant chemotherapy after fertility preservation. Many HR-positive patients received adjuvant endocrine therapy.	Performed before gonadotoxic therapy.	Controlled ovarian stimulation with gonadotropins, often plus letrozole in ER-positive patients, with oocyte and embryo cryopreservation.	Good ovarian response observed; several women returned for embryo transfer, with reassuring live birth rates.	Median oncologic follow-up (5 years)	Fertility preservation before chemotherapy is feasible and does not appear to compromise oncologic outcomes. Long-term data support the safety of COS with letrozole in young breast cancer patients.
Azim et al., 2024 [30]	International, multicenter, single-arm, prospective trial (secondary analysis of POSITIVE) *	516	Women with hormone receptor-positive breast cancer and BRCA	All patients received adjuvant endocrine therapy (ET) for 18–30 months before study entry	Fertility preservation performed before chemotherapy.	ART and cryopreservation	ART outcomes: Cryopreserved embryo transfer increased pregnancy odds (OR 2.41); IVF, IUI, and clomiphene donation were not significant. Menstruation recovery: 85% by 6 mo, 94.2% by 12 mo; age not a significant predictor (35–39 OR 0.50, 40–42 OR 0.16).	Median Follow-up: 41 months	1. High Pregnancy Rates: 74% achieved pregnancy; young age is the main determinant of shorter time to pregnancy. 2. Cryopreservation Works Best: Embryo/oocyte cryopreservation at diagnosis, followed by later embryo transfer, was associated with significantly higher pregnancy rates (OR 2.41). 3. No Short-Term Safety Signal: Ovarian stimulation for FP at diagnosis was not associated with increased short-term (3-year) risk of recurrence, even in hormone receptor-positive disease.
**GnRH Agonist Studies**									
Lee et al., 2020 [31]	Prospective cohort study.	67	ER– Breast Cancer Patients	Cyclophosphamide-based chemotherapy (most common regimen: doxorubicin and cyclophosphamide followed by taxane). Mean cumulative cyclophosphamide dose was 3841.9 +/– 432.2 gram	GnRH agonists were administered starting before chemotherapy.	Ovarian protection with GnRHa	Success defined as resumption of menstruation AND serum AMH > 1 ng/mL at 12 months after chemotherapy. Menstruation Resumption: 97% (65/67) of women. AMH > 1 ng/mL: 70.1% (47/67) of women	12 months after the completion of chemotherapy.	Higher AMH associated with better GnRH agonist response
Zhong et al., 2019 [32]	Randomized controlled study	98	Premenopausalwomen, aged 18 to 45 years (median age: 39.0 years); HER-2+/– ER+/–	Adjuvant anthracycline-based chemotherapy (100%); 79% received additional taxane; HR+ patients received toremifene.	GnRHa administration was concurrent with chemotherapy, starting within 1 week of the initial dose.	Ovarian protection with GnRHa	Primary outcome: OVF (amenorrhea ≥ 6 months) at 1 year; 80.6% chemo alone vs. 44.7% chemo + goserelin (*p* = 0.002).	Median follow-up time was 15 months. Primary end point (OVF) was assessed at 1 year after chemotherapy.	GnRHa protects ovarian function during chemotherapy regardless of HR status; AMH predicts chemotherapy-induced ovarian failure.
**IVM/OTC Studies**									
Arab et al., 2022 [33]	retrospective cohort study	132	(1) women diagnosed with breast cancer at <35 years old; TNBC, ER, PR, HER2, BRCA, BRCA1, BRCA2	Not applicable; FP performed before gonadotoxic therapy.	Performed before gonadotoxic therapy.	IVF, IVM.	Baseline FSH/AFC similar; hereditary group had more cryopreserved embryos (3.35 vs. 1.9, *p* = 0.046) and lower ER/HER2 positivity (7.5% vs. 32%).	The study does not state a specific follow-up duration for patients. The only follow-up data reported is regarding return rates for fertility treatment.	77.5% of mutations were BRCA; hereditary and non-hereditary patients had similar ovarian stimulation response; higher embryo yield in hereditary patients likely due to younger age (30.7 vs. 32.4 yrs, *p* = 0.034).
Grynberg et al., 2019 [34]	Retrospective cohort study	329	Mean age ~32.1 years, premenopausal al. All invasive ductal carcinoma; BRCA, BRCA1, BRCA2, ER, PR	All candidates for neoadjuvant chemo (regimens not detailed)	Before starting neoadjuvant chemotherapy	In vitro maturation (IVM) of oocytes.	Findings: No difference in AFC, AMH, oocytes retrieved, or IVM maturation rates between BRCA+ and BRCA groups.	Not applicable (single procedure).	BRCA1/2 gene mutations do not affect the capacity of oocytes from breast cancer candidates for fertility preservation to mature in vitro.
Raad et al., 2022 [35]	Retrospective cohort study Single center	321	Women aged 18–41 y with invasive breast cancer, indicated for neoadjuvant chemotherapy, no prior chemo or current hormone therapy, two ovaries, and ≥10 small antral follicles; triple-negative status, HER2 +/− overexpression,	Planned neoadjuvant chemotherapy, fertility preservation performed before chemotherapy No prior gonadotoxic treatment	Performed urgently before initiation of neoadjuvant chemotherapy. IVM was scheduled 1–3 days after oncofertility counseling. Retrieval during the follicular (63%) or luteal (37%) phase.	Oocyte vitrification after IVM (in vitro maturation). Immature oocyte retrieval without ovarian stimulation. 24–48 hours in vitro maturation. Mature (MII) oocytes vitrified. Ovarian tissue cryopreservation (OTC) was offered in combination (61 patients).	AMH levels were significantly lower in the case of triple-negative BC when compared with ER/PR/HER2 status-positive cancer; Multivariate statistical analysis showed that HER2-positive status was associated with a mean maturation rate < 60%	Outcomes assessed per IVM cycle. No reproductive follow-up (pregnancy/live birth).	triple-negative BC, and HER2 overexpression may negatively influence IVM outcomes. HER2 is independent predictors of poor IVM outcomes. AMH < 1.5 ng/mL predicts lower maturation rates. Tumor aggressiveness may affect ovarian function and folliculogenesis. Important implications for FP counselling in urgent breast cancer cases.

* Although the POSITIVE trial is not a conventional cohort study, this secondary analysis employs a prospective cohort-type design and reports subtype-stratified fertility preservation and reproductive outcomes in premenopausal breast cancer patients, satisfying the inclusion criteria for study design, population, and outcomes. It was therefore included in the final synthesis.

## Data Availability

The data presented in this study are available on request from the corresponding author.

## Abbreviations

AFC	antral follicle count
AI	aromatase inhibitor
AMH	anti-Müllerian hormone
ART	assisted reproductive technology
ASCO	American Society of Clinical Oncology
BC	breast cancer
BCFI	breast cancer-free interval
BMI	body mass index
BRCA	breast cancer susceptibility gene
CDK4/6	cyclin-dependent kinase 4/6
COC	cumulus-oocyte complex
COS	controlled ovarian stimulation
DFS	disease-free survival
E2	estradiol
ER	estrogen receptor
ESMO	European Society for Medical Oncology
ET	endocrine therapy
FEC	5-fluorouracil, epirubicin, cyclophosphamide
FP	fertility preservation
FSH	follicle-stimulating hormone
GnRH	gonadotropin-releasing hormone
GnRHa	gonadotropin-releasing hormone agonist
HER2	human epidermal growth factor receptor 2
HR	hormone receptor
ICSI	intracytoplasmic sperm injection
IUI	intrauterine insemination
IVF	in vitro fertilization
IVM	in vitro maturation
LH	luteinizing hormone
MII	metaphase II
NAC	neoadjuvant chemotherapy
OFS	ovarian function suppression
OTC	ovarian tissue cryopreservation
OVF	ovarian function failure
PARP	poly ADP-ribose polymerase
PR	progesterone receptor
RCT	randomized controlled trial
SBR	Scarff-Bloom-Richardson
SERM	selective estrogen receptor modulator
TNBC	triple-negative breast cancer
